# Portuguese Propolis Antitumoral Activity in Melanoma Involves ROS Production and Induction of Apoptosis

**DOI:** 10.3390/molecules27113533

**Published:** 2022-05-31

**Authors:** Rafaela Dias Oliveira, Sónia Pires Celeiro, Catarina Barbosa-Matos, Ana Sofia Freitas, Susana M. Cardoso, Marta Viana-Pereira, Cristina Almeida-Aguiar, Fátima Baltazar

**Affiliations:** 1 CBMA−Centre of Molecular and Environmental Biology, Department of Biology, University of Minho, 4710-057 Braga, Portugal; rafaeladiasoliveira98@gmail.com (R.D.O.); anasofiapfreitas@gmail.com (A.S.F.); 2 Life and Health Sciences Research Institute (ICVS), School of Medicine, University of Minho, Campus of Gualtar, 4710-057 Braga, Portugal; sonia.celeiro94@gmail.com (S.P.C.); ccatarinabm@gmail.com (C.B.-M.); martavianap@gmail.com (M.V.-P.); 3 ICVS/3B’s-PT Government Associate Laboratory, 4710-057 Braga/4806-909 Guimarães, Portugal; 4 LAQV-REQUIMTE & Department of Chemistry, University of Aveiro, 3810-193 Aveiro, Portugal; susanacardoso@ua.pt

**Keywords:** Portuguese propolis, cancer, melanoma, antitumoral activity, antioxidant activity, pro-oxidant activity, apoptosis

## Abstract

Melanoma is the most aggressive and life-threatening skin cancer type. The melanoma genome is the most frequently mutated, with the *BRAF* mutation present in 40–60% of melanoma cases. *BRAF*-mutated melanomas are characterized by a higher aggressiveness and progression. Adjuvant targeted treatments, such as BRAF and MEK inhibitors, are added to surgical excision in *BRAF*-mutated metastatic melanomas to maximize treatment effectiveness. However, resistance remains the major therapeutic problem. Interest in natural products, like propolis, for therapeutic applications, has increased in the last years. Propolis healing proprieties offer great potential for the development of novel cancer drugs. As the activity of Portuguese propolis has never been studied in melanoma, we evaluated the antitumoral activity of propolis from Gerês (G18.EE) and its fractions (*n*-hexane, ethyl acetate (EtOAc), and *n*-butanol) in A375 and WM9 melanoma cell lines. Results from DPPH•/ABTS• radical scavenging assays indicated that the samples had relevant antioxidant activity, however, this was not confirmed in the cell models. G18.EE and its fractions decreased cell viability (SRB assay) and promoted ROS production (DHE/Mitotracker probes by flow cytometry), leading to activation of apoptotic signaling (expression of apoptosis markers). Our results suggest that the *n*-BuOH fraction has the potential to be explored in the pharmacological therapy of melanoma.

## 1. Introduction

Cancer is the world’s second-biggest cause of death, with approximately 10 million deaths registered in 2020. Each year, the number of new cases grows at an exponential rate, with over 19.3 million cases reported in 2020 [1]. Skin cancer is a very common malignancy that can be divided into three major types: basal cell carcinoma (BCC), squamous cell carcinoma (SCC), and cutaneous malignant melanoma (CM) [2,3]. This classification is based on clinical behavior and the origin of the cells that give rise to skin cancer. BCC and SCC are non-melanoma skin cancers and are relatively curable, with a high occurrence rate [4,5]. Melanoma is caused by the uncontrolled proliferation of melanocytes [6] and is the most aggressive, life-threatening, and invasive form of skin cancer [6,7]. Its incidence has increased steadily and significantly in recent years, primarily among white populations [6]. According to the International Agency for Research on Cancer (IARC), 324,635 new melanoma cases and 57,043 patient deaths were reported in 2020 [1,8]. When diagnosed at early stages, melanoma has a 5-year survival rate higher than 90%, and it can be successfully treated with surgery alone [9,10]. However, patients eventually acquire metastases as a result of the rapid disease progression and dissemination, decreasing the 5-year survival rate to 15% [11]. Treatment for advanced-stage melanoma patients includes surgical excision, targeted therapies, and immunotherapies [12].

Melanoma genomes have the highest mutation rate, with V-RAF murine sarcoma viral oncogene homolog B (*BRAF*) mutations being the most prevalent, which occur in roughly 40–60% of melanoma cases [13]. More than 90% of these mutations appear at codon 600 of the *BRAF* gene (*BRAF^600E^*), which leads to the substitution of valine by glutamic acid, resulting in hyperactivation of the MAP kinase/ERK signaling pathway. This pathway hyperactivation is interconnected to basic cellular processes like proliferation, migration, apoptosis, and tumor growth. *BRAF*-mutated melanomas exhibit a high level of aggressiveness and progression, being more predisposed to metastasize, especially to the brain [14,15]. Specific BRAF and MEK inhibitors are currently used in melanoma therapy to increase survival in *BRAF*-mutated patients.

BRAF and MEK targeted therapies substantially improved the *BRAF* mutated patients’ outcomes, and the combination of these targeted inhibitors is currently the gold standard treatment for high-risk resected melanoma patients. However, primary and acquired resistance to these treatments remains a major health problem [16,17]. Regarding all this evidence, research focused on alternative medicines, such as phytochemicals from plants and natural extracts, which offer great potential for therapeutic innovation. In fact, a high number of natural compounds have demonstrated immunomodulatory and anticancer activity, creating favorable conditions for the development of novel treatments [7,18].

Propolis, or “bee glue”, is a honeybee-made brownish resinous and balsamic product (mainly produced by *Apis mellifera* L.) obtained by mixing resin collected from several plants’ buds and exudates with beeswax, pollen, and salivary enzymes [19,20]. Etymologically, propolis comes from the Greek words “pro” and “polis”, which mean “in defense” and “city”, respectively, revealing its function in hive protection [19]. The chemical composition of this natural product is very complex and highly variable, depending on several factors, namely phytogeographical origin, the harvesting time and techniques, and the solvent extraction and the extraction method [20,21,22]. One of the major struggles with the use of propolis as a therapeutic agent in conventional medicine, particularly in the health system, is the absence of chemical standardization [23,24]. More than 500 compounds were already identified on propolis samples, including phenolic acids, flavonoids, esters, diterpenes, sesquiterpenes, lignans, aromatic aldehydes, alcohols, amino acids, fatty acids, vitamins, and minerals [22,25,26,27].

Propolis is a non-toxic material with a high number of biological, pharmacological, and biomedical properties, such as immunostimulant [28], antibacterial [29], antifungal [30,31], anti-inflammatory [32], antiviral [33,34], antioxidant [35,36], antitumor/anticancer [20,32,37], anesthetic [19,38], cariostatic [39], antiprotozoal [24], antihypertensive [40], anti-hepatotoxic/hepatoprotective [35], antineurodegenerative [41], antituberculosis [42], radioprotective [43], genotoxic and anti-genotoxic [22]. The growing interest in biological information is supported by the positive impact of these biological properties on human health [44,45]. One of the most well-known propolis bioactivities is antioxidant activity. Propolis antioxidant properties have been correlated with its total polyphenol content [46] and flavonoids [35,47]. CAPE, a common component of poplar propolis, seems to be one of the most powerful antioxidant compounds [48]. Portuguese propolis, according to Valente et al., has a protective effect on human erythrocytes, shielding them from damage caused by reactive oxygen species (ROS). This suggests that this type of propolis is an important and powerful antioxidant agent that can be used against oxidative stress, being beneficial for human health [37].

Furthermore, the increasing number of studies related to the cytotoxicity of propolis and its constituents in vitro and with antitumor activity in vivo showed that this natural product could be a promising antitumoral agent [20,49,50]. Propolis and its phenolic compounds exhibit a protective effect against different cancer types, including breast, colon, uterine, cervix, lung, pancreatic, skin (including melanoma), and renal, among others [51,52]. Briefly, this natural product can inhibit specific oncogene signaling pathways (e.g., β-catenin, c-myc, NF-κB, and some intermediates of the PI3K/AKT pathway), resulting in a reduction in cell proliferation and growth. It can also act by reducing the cancer stem cell population, increasing apoptosis, exerting antiangiogenic effects, and modifying the tumor microenvironment [50]. Propolis from Portugal has been demonstrated to have anticancer efficacy against malignancies such as breast [20], renal cell carcinoma [37], and human colorectal cancer [20,49]. However, its activity against melanoma is unknown at this point.

Research on Portuguese propolis has contributed to increasing the value of this natural beehive product, opening new perspectives for the development of new propolis-based therapies with a positive impact on human health. Therefore, in the present study, the antitumor activity of a Portuguese propolis sample collected in an apiary located in Gerês, Portugal, was evaluated on A375 and WM9 melanoma cell lines. To the best of our knowledge, this is the first time that Portuguese propolis—in this case from Gerês—has been investigated for its anticancer potential in melanoma cells.

## 2. Results

### 2.1. Chemical Composition of G18.EE and Its Fractions

The ethanol extract of propolis from Gerês harvested in 2018 (G18.EE) had a total phenolic content (TPC) of 224.60 ± 10.86 mg/g extract and, according to the UPLC-DAD-ESI/MS^n^ analysis, it was rich in the flavonoids apigenin, pinobanksin, chrysin, acacetin, galangin, kaempferide, kaempferol, and kaempferol–methoxy-methyl ether, and also in phenolic acids and its derivates, including caffeic acid, caffeic acid isoprenyl ester (CAIE), 3,4-dimethyl-caffeic acid (DMCA), *p*-coumaric acid, *p*-coumaric acid methyl ester, and ferulic acid (Supplementary Figure S1 and Supplementary Table S1).

Propolis fractions were obtained by sequential fractionation of the crude extract with *n*-hexane, EtOAc, and *n*-BuOH. Apart from the *n*-hexane fraction that showed non-defined chromatographic peaks (possibly due to the lower levels of phenolic compounds and to co-elution with non-phenolic compounds that may appear as dominant constituents in this sample), EtOAc and *n*-BuOH were both characterized by the presence of apigenin, pinobanksin, pinobanksin 3-*O*-acetate, chrysin, acacetin, CAIE, and pinocembrin. The levels of the main phenolic components of these fractions were higher than in G18.EE (superior total area of their corresponding chromatographic peaks, Supplementary Figure S1 and Supplementary Table S1). There were still some clear differences between EtOAc and *n*-BuOH as well—the first was rich in phenolic acids and their derivates, such as caffeic acid, 3,4-dimethyl-caffeic acid (DMCA), and *p*-coumaric acid, while these components were not prominent in the *n*-BuOH fraction. Taking these characteristics into account, we can assume that the EtOAc fraction is the most identical to G18.EE.

CAPE, a common component of poplar propolis, presents an important antitumor activity [20,48]. CAPE was found in the G18.EE and its three fractions ([M-H]^−^ at *m*/*z* 283, peak 22; Supplementary Figure S1 and Supplementary Table S1), however, with distinct abundances, which follow the order *n*-BuOH ≥ EtOAc > G18.EE > *n*-hexane.

### 2.2. G18.EE Displays Radical Scavenging Activity

To evaluate the free-radical scavenging activity of the G18 ethanol extract, we used a methodology based on the reduction of a stable free radical, the DPPH•. The value of EC_50_ (concentration that produces half of the maximal response) determined for the G18.EE was 10.90 ± 0.34 µg/mL (Table 1). According to Sheng et al. [51], a natural substance can be identified as a possible natural antioxidant if it exhibits DPPH• scavenging activity. As a result, we can deduce that G18.EE may be a potential natural antioxidant. Gallic acid is used as a conventional standard for DPPH assay, and its EC_50_ value was 1.21 ± 0.08 µg/mL.

The 2,2′-azino-bis (3-ethylbenzothiazoline-6-sulfonic acid) (ABTS) assay was also used to assess the antioxidant activity of G18.EE. ABTS assay uses the absorbance of the ABTS• colored radical cation to measure a compound’s antioxidant capacity. Trolox was used as standard, and the EC_50_ value for G18.EE was 9.83 ± 0.21 µg/mL (Table 1). These findings support the previous results of the DPPH assay.

### 2.3. Antitumoral Activity of G18.EE and Its Fractions on Melanoma Cells

To evaluate the melanoma antitumoral potential of G18.EE and its fractions, we first determined the half-maximal inhibitory concentration (IC_50_), the 25% inhibition concentration (IC_25_), and the 15% inhibition concentration (IC_15_) for each propolis sample and cell line.

#### 2.3.1. G18.EE and Its Fractions Decreased Melanoma Cell Viability in a Dose-Dependent Manner

**Figure 1:**
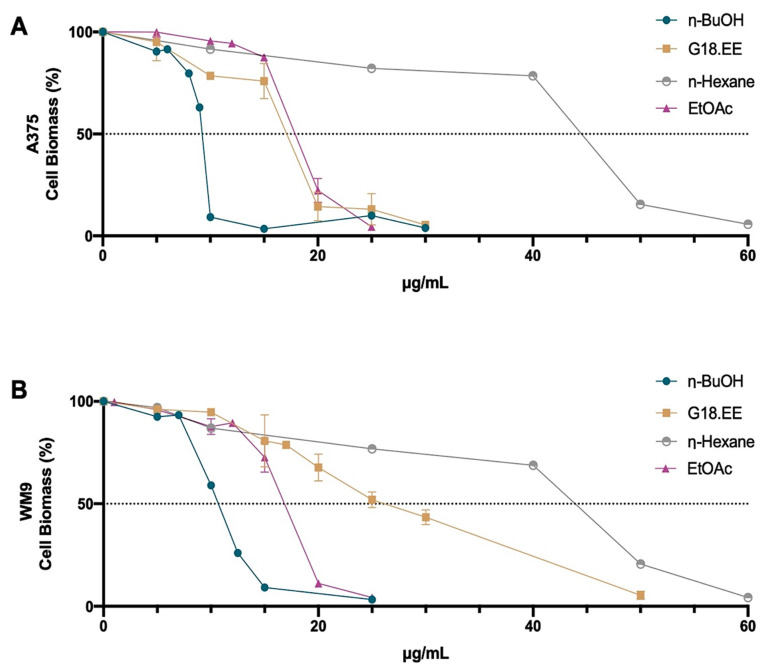
Effect of G18.EE and its fractions on total cell biomass of melanoma cells. A375 (**A**) and WM9 (**B**) cell lines were treated with a range of concentrations (5 to 60 µg/mL) of propolis extract (G18.EE) and its fractions (*n*-hexane, EtOAc, and *n*-butanol) for 72 h. Cell biomass was measured by the Sulphorhodamine B (SRB) assay. Data were normalized for total biomass. Results represent the mean ± SD of three independent experiments carried out in triplicate.

The cytotoxic effect of G18.EE and its fractions were evaluated in A375 and WM9 human melanoma cell lines through the Sulphorhodamine B (SRB) assay. In Figure 1, it is possible to observe a decrease in cell biomass after a 72 h (h) treatment with G18.EE and its fractions (concentrations ranging from 5 to 60 μg/mL) in a dose-dependent manner. It is also possible to infer that the viability of the melanoma cell lines is affected differently by the various fractions tested. A375 cells (Figure 1A) appear to be more sensitive to G18.EE, *n*-BuOH, and EtOAc fractions, whereas WM9 cells (Figure 1B) are more sensitive to *n*-BuOH and EtOAc. The *n*-hexane fraction was the least active and *n*-BuOH the most active fraction against both melanoma cell lines tested.

The values of IC_50_, IC_25_, and IC_15_ (Table 2) were calculated through the curve obtained in Figure 1. For subsequent studies, we chose the two treatments with the lowest IC_50_ value for each cell line: *n*-BuOH and G18.EE for A375 cells; *n*-BuOH and EtOAc for WM9 cells. As previously mentioned, *n*-Hexane was the fraction with the lowest toxicity for both cell lines.

#### 2.3.2. Melanoma Cell Viability for IC_15_ and IC_25_ Intermediate Concentrations of G18.EE and Its Fractions

**Figure 2:**
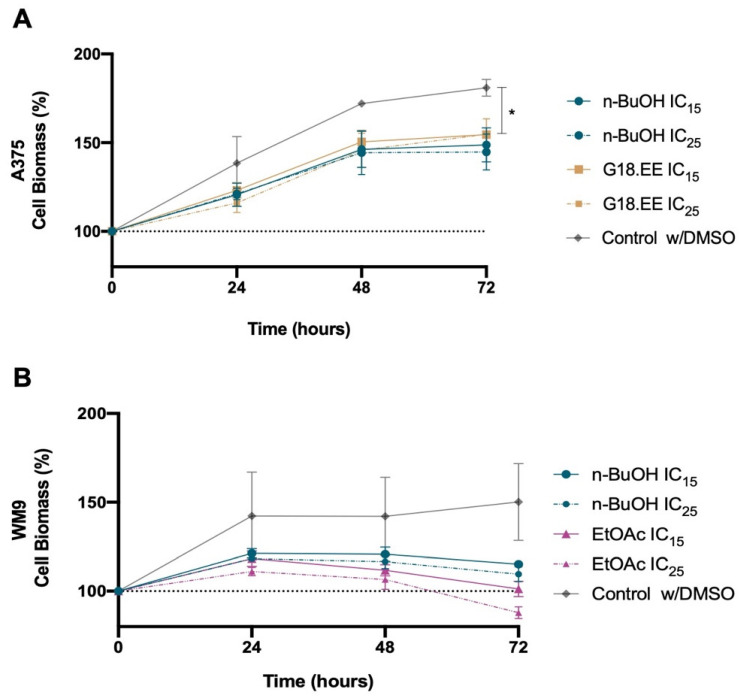
Effect of the two selected concentrations of propolis fractions on cell biomass (A375 and WM9 cells) over time. Cell biomass was measured at 24, 48, and 72 h by SRB assay after treatment with (**A**) 5 µg/mL (IC_15_) and 7 µg/mL (IC_25_) *n*-BuOH and 10 µg/mL (IC_15_) and 13 µg/mL (IC_25_) G18.EE for A375 cell line; (**B**) 5 µg/mL (IC_15_) and 7 µg/mL (IC_25_) *n*-BuOH and 8 µg/mL (IC_15_) and 10 µg/mL (IC_25_) EtOAc for WM9 cell line. A375 and WM9 cells treated with DMSO were used as controls. Results are expressed as mean ± SD. Three independent experiments were carried out in triplicate. * *p* < 0.05.

Instead of using the IC_15_ and IC_25_ concentrations of each fraction for each cell line (Table 2), we selected an intermediate concentration of each fraction for both cell lines for the following assays (Table 3). For example, the IC_25_ values of *n*-butanol for A375 and WM9 cells were 6.16 µg/mL and 8.08 µg/mL, respectively. Therefore, we chose 7 µg/mL as the IC_25_ value for *n*-BuOH. The cytotoxicity of these IC_15_ and IC_25_ concentrations was assessed by SRB assay over time (Figure 2).

The viability assay over time shows that G18.EE, *n*-BuOH, and EtOAc, even at lower doses, have an impact on melanoma cell viability at the initial time points (Figure 2). Statistical analyses verified if these lower doses affected melanoma cell biomass over time and independently. There was no statistically significant interaction between the effects of treatment on cell biomass over time in the A375 cell line (*p* = 0.5901; Supplementary Table S2). Simple main effects analysis showed, however, that time and treatment independently have a significant influence on cell biomass (*p* < 0.0001; *p* = 0.0205; respectively; Supplementary Table S2). Respective multiple comparisons to the control demonstrated that G18.EE IC_15_ has a significant effect on A375 cell biomass at 72 h (*p* = 0.0406; Figure 2A). Regarding the WM9 cell line, a statistically significant interaction was verified between the effects of treatment and time (*p* = 0.0074). Time has an independent statistically significant influence (*p* = 0.0013), whereas treatment did not significantly affect cell biomass (*p* = 0.1252) (Supplementary Table S2).

#### 2.3.3. G18.EE and Its Fractions (*n*-BuOH and EtOAc) Promote Mitochondrial ROS Production in Melanoma Cells

**Figure 3:**
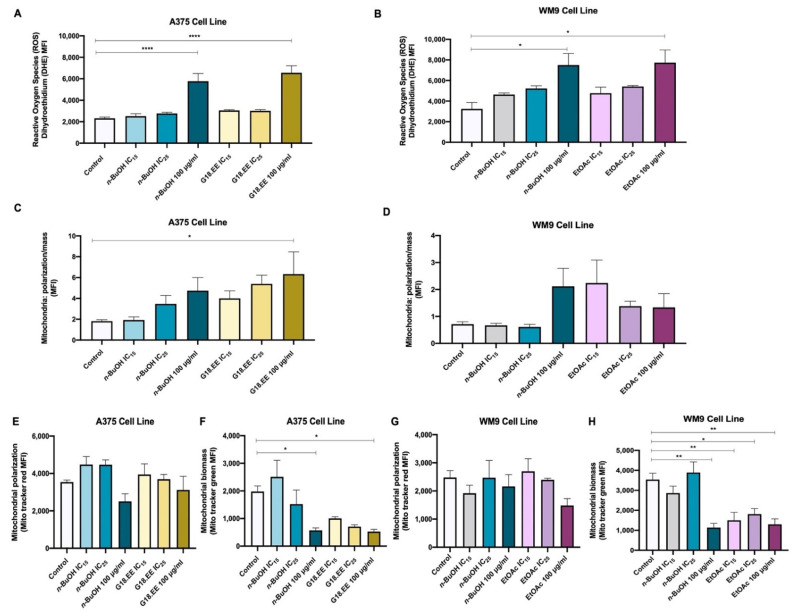
Treatments with G18.EE and its fractions (*n*-BuOH and EtOAc) increase ROS levels and mitochondrial activity. Results were obtained after 24 h treatment with DMSO (control); *n*-BuOH 5 µg/mL (IC_15_), 7 µg/mL (IC_25_), and 100 µg/mL; and G18.EE 10 µg/mL (IC_15_), 13 µg/mL (IC_25_), and 100 µg/mL. ROS levels were measured in A375 (**A**) and WM9 (**B**) cell lines. Mitochondrial activity was measured in (**C**) A375 and (**D**) WM9 cell lines through the ratio of the respective (**E**,**G**) mitochondrial polarization by the (**F**,**H**) mitochondrial biomass. Results are expressed as mean ± SD. Three independent experiments were carried out in triplicate. * *p* < 0.05, ** *p* < 0.01, **** *p* < 0.0001.

ROS production was evaluated to verify if the selected treatments—*n*-BuOH and G18.EE for A375, and *n*-BuOH and EtOAc for WM9—had an antioxidant effect on melanoma cell lines (Figure 3), as expected based on the DPPH• and ABTS• scavenging activity results (Table 1). In the DPPH and ABTS assays, 150 µg/mL of propolis was used. To compare with these assays, 100 µg/mL of each fraction was the dose used in the ROS assays. However, results show that the different treatments increased ROS production in melanoma cell lines in a dose-dependent manner (Figure 3A,B).

The different treatments had a significantly influence in the ROS levels (*p* < 0.0001; *p* = 0.0157, respectively; Supplementary Table S3) in both cell lines. Dunnett’s test for multiple comparisons demonstrates that 100 µg/mL of *n*-BuOH and G18.EE were significantly different than the control condition (DHE) (*p* < 0.0001; *p *< 0.0001; Figure 3A). Regarding WM9 cells, the same multiple comparison tests revealed that 100 µg/mL of *n*-BuOH and EtOAc significantly increased ROS levels compared to the control (*p* = 0.0072; *p* = 0.0047; Figure 3B).

Since mitochondria are the primary source of intracellular ROS [53], the activity of this cell organelle was measured to understand if the greater levels of ROS observed (Figure 3A,B) are explained by a higher mitochondrial activity (Figure 3C,D). Mitochondrial activity was assessed through the ratio of mitochondrial polarization: mitochondrial mass. Although no statistically significant differences were detected between treatments, these seem to be associated with higher mitochondrial activity in both cell lines (Supplementary Table S4). In the A375 cells multiple comparisons test, mitochondrial activity was significantly higher with 100 µg/mL of G18.EE, when compared to the control condition (*p* = 0.0460; Figure 3C). In WM9 cells, 100 µg/mL of either *n*-BuOH or EtOAc seems to induce an increase in mitochondrial activity compared to the control. However, the results were not statistically significant (Figure 3D).

A significant impact of G18.EE and its selected fractions (*n*-BuOH and EtOAc) was observed in the mitochondrial biomass of both cell lines (*p* = 0.0028; *p* = 0.0002, respectively; Supplementary Table S5; Figure 3F,H); 100 µg/mL of *n*-BuOH and G18.EE in the A375 cell line significantly decreased the biomass of this organelle (*p* = 0.0306 and *p* = 0.0253, respectively; Figure 3F). In WM9 cells, 100 µg/mL of *n*-BuOH and IC_15_, IC_25_ and 100 µg/mL of EtOAc decreased mitochondrial biomass (*p* = 0.0013, *p* = 0.0051, *p* = 0.0168, and *p* = 0.0024; Figure 3H).

#### 2.3.4. G18.EE, *n*-BuOH, and EtOAc Treatments Induce Apoptosis in Melanoma Cell Lines

**Figure 4:**
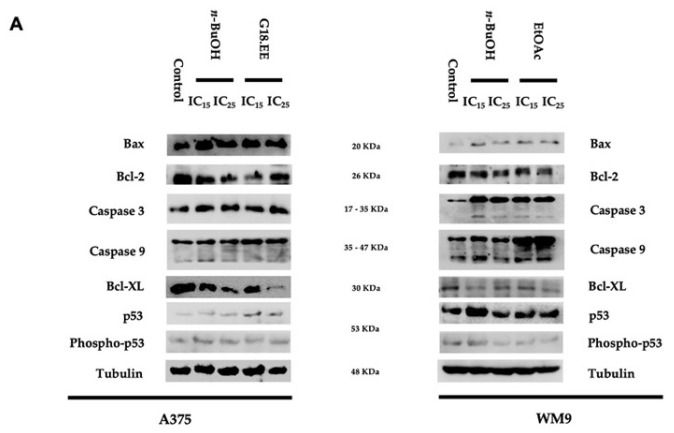

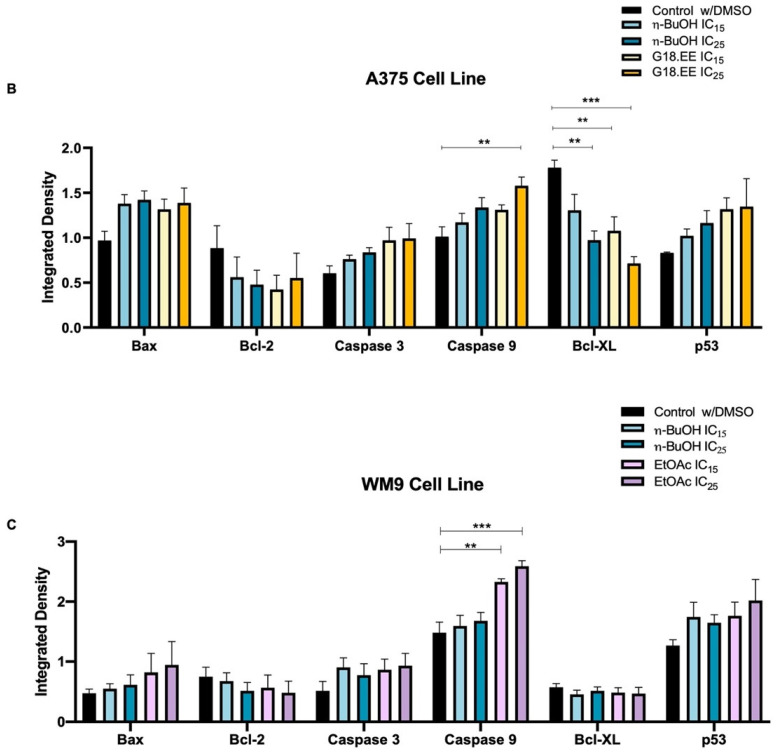
Pro-apoptotic mechanisms are activated by G18.EE, *n*-BuOH, and EtOAc in melanoma cells. Apoptotic markers were evaluated in the different conditions by (**A**) Western blot and quantified in A375 (**B**) and WM9 (**C**) cell lines. A375 and WM9 cells treated with DMSO were used as controls. Except for phospho-p53, which was normalized for total p53, the remaining proteins were normalized to tubulin. Results are expressed as mean ± SD. Results are from three independent experiments carried out in triplicate. ** *p* < 0.01, *** *p* < 0.001.

ROS overproduction can stimulate tumor cell apoptosis [54]. Therefore, the levels of apoptotic markers were evaluated to understand if the different propolis treatments induced this cell death mechanism (Figure 4). The levels of anti-apoptotic, Bcl-2, and Bcl-XL; pro-apoptotic, Bax, and p53; and apoptotic-related proteins, such as Caspase 3 and Caspase 9, were evaluated (Figure 4A). The results suggest that Gerês propolis treatments trigger cell death by a regulated cell death mechanism. Except for Bcl-XL in WM9 cells, anti-apoptotic Bcl-2 and Bcl-XL proteins appear to be reduced in the different treatment conditions (Figure 4B,C). Both cell lines tested seem to display higher levels of Bax and p53, although not significant, which are known pro-apoptotic indicators (Figure 4B,C). The apoptotic proteases caspases 3 and 9 also appear to be enhanced by propolis treatments (Figure 4B,C).

Propolis extract and its fractions significantly affected caspase 9 expression levels in A375 (*p* = 0.0201) and WM9 cells (*p* = 0.0006) as well as the Bcl-XL levels in A375 (*p* = 0.0014) cell line (Figure 4B,C; Supplementary Table S7). More specifically, the levels of caspase 9 were significantly increased with G18.EE IC_25_ (*p* = 0.0063) in A375 cell line, when compared to the control; and with EtOAc IC_15_ (*p* = 0.0046) and IC_25_ (*p* = 0.0007) in WM9 cell line (Figure 4B,C). In the A375 cell line, Bcl-XL was significantly lower with η-BuOH IC_15_ (*p* = 0.0035), G18.EE IC_15_ (*p* = 0.0087), and IC_25_ (*p* = 0.0005) treatments (Figure 4B).

## 3. Discussion

Melanoma is the most aggressive, severe, and invasive type of skin cancer [6,7]. Despite all the advances and research in melanoma treatment, finding effective therapies remains a challenge. The current therapeutic dilemma is caused by acquired resistance and the side effects of traditional medicines [18]. Due to the drawbacks of standard treatments, natural products, such as propolis or its isolated compounds, have the potential to be added to the therapeutic arsenal of cancer [7,18]. Propolis antitumor bioactivity has been described in some types of cancer, including skin cancer. However, results using Portuguese propolis in melanoma are lacking. Therefore, in this study, we evaluated, for the first time, the therapeutic potential of G18.EE and its fractions on *BRAF*-mutated melanoma cells.

Propolis presents a very complex chemical composition, being composed of a high number of phenolic compounds. Flavonoids and phenolic acids are the major classes identified in this natural product [20,55]. UPLC-DAD-ESI/MS^n^ analysis showed apigenin, pinobanksin, chrysin, acacetin, galangin, kaempferide, kaempferol, and kaempferol–methoxy-methyl ether, caffeic acid, caffeic acid isoprenyl ester (CAIE), 3,4-dimethyl-caffeic acid (DMCA), *p*-coumaric acid, *p*-coumaric acid methyl ester, and ferulic acid as the most prevalent components of G18.EE (Supplementary Figure S1 and Supplementary Table S1). These main phenolic compounds correspond to those described in other Portuguese and European propolis samples [21,56,57,58], and in particular with those from the same harvesting location. In fact, as evidenced by Freitas et al., for propolis samples collected between 2011 and 2014 in Gerês, phenolic constituents were maintained, although there were variations in their abundance [36].

Considering that DPPH• is a stable nitrogen-centered free radical, substances that can react with it, leading to a change of color from purple to yellow, are called antioxidants and are, therefore, antiradical agents [58]. In the case of ABTS• scavenging assay, the compounds have antioxidant capacity if a reduction of ABTS• was verified by decolorization [59]. Previous studies classified Portuguese propolis as a natural product with antioxidant activity [60]. Falcão et al. [61] verified that propolis samples from the north and coast of Portugal, as well as from the Azores, have the best DPPH• scavenging effects, with EC_50_ values ranging from 10–30 µg/mL when compared to propolis from other zones of Portugal. In particular, the sample collected in Montalegre (north of Portugal) exhibited a scavenging activity effect with an EC_50_ value of 10 µg/mL. According to Freitas et al. [62], G18.EE has an EC_50_ value of 12.40 ± 0.43 µg/mL. This value was calculated in 2018, the sample’s harvest year. In this study, the EC_50_ values calculated for G18.EE were 10.90 ± 0.34 (DPPH assay) and 9.83 ± 0.21 µg/mL (ABTS assay) (Table 1), indicating that the antioxidant capacity of Gerês (2018) Portuguese propolis was preserved over the years. According to the data published by Da Cruz et al., the EC_50_ of G18.EE is much lower than other values published for worldwide samples, implying that it has stronger DPPH• scavenging activity [63]. As an outcome, G18.EE is an attractive type of propolis for antioxidant applications.

Regarding the antitumor activity of Portuguese propolis in *BRAF*-mutated melanoma cells, our results reveal that G18.EE and its fractions were able to reduce A375 and WM9 cell viability. *n*-Hexane was the least active fraction (Figure 1; Table 2), and *n*-BuOH was the most cytotoxic propolis fraction for both melanoma cell lines. These findings corroborate prior research that identified other propolis samples as cytotoxic agents in melanoma, such as Moroccan [64] and Algerian [65], among others. CAPE is a propolis component that presents important anticancer and chemoprotective activities [20,48]. This compound was identified in G18.EE and its fractions (Supplementary Figure S1 and Supplementary Table S1) using UPLC-DAD-ESI/MSn analysis, presenting higher levels in the *n*-BuOH fraction and a lower value of total area in the *n*-hexane fraction. This supports the results from the cell viability assay, suggesting that CAPE can be a bioactive compound against melanoma cells. Since *n*-BuOH/G18.EE and *n*-BuOH/EtOAc were the most active fractions for A375 and WM9 cells, respectively. We tested the in vitro cytotoxic effect of IC_15_ and IC_25_ concentrations of these treatments (Table 3). The different fraction treatments, even at lower doses, seem to affect melanoma cell viability at early time points (Figure 2).

According to data from the DPPH and ABTS scavenging assays, propolis exhibits antioxidant capacity. However, this bioactivity of propolis has not been validated in cells. According to Cisilotto and collaborators, in the SK-MEL-28 *BRAF*-mutated melanoma cell line, Brazilian propolis increased ROS accumulation, demonstrating that propolis had a pro-oxidant function [66]. Our DHE data support this tendency for increased ROS levels in propolis-treated cells. In A375 and WM9 cell lines, ROS production was enhanced in a dose-dependent manner (Figure 3A,B). Since mitochondria are the primary source of ROS [53], we decided to investigate propolis effects on mitochondrial activity. The results suggest that treatment with propolis extract and its fractions is associated with higher mitochondrial activity (Figure 3C,D) but lower mitochondrial biomass (Figure 3F,H). Oxidative stress, which is characterized by an overproduction of ROS, can induce mitochondrial alterations and damage [67], which could explain the reduced biomass seen in treated A375 and WM9 cells. Based on these findings, we suggest that the commonly used DPPH assay to measure propolis scavenging activity does not correctly reflect what happens in the cancer context. In fact, our research showed that relying on scavenging assays, such as DPHH and ABTS, to claim that propolis has antioxidant capabilities can be misleading.

In vitro antioxidant activity of propolis from Gerês (G11, G12, G13, and G14) was observed by Freitas et al. [36] through a DPPH scavenging assay (EC_50_ range from 14.41 ± 0.56 to 25.24 ± 2.45 µg/mL). These results were supported by cytometry data employing *Saccharomyces cerevisiae* as a biological model, which demonstrated that propolis from Gerês (concentrations tested ranging from 50 to 200 µg/mL) decreases intracellular oxidation triggered by H_2_O_2_, the most prevalent ROS in vivo [36]. Propolis has been described as having opposing activities, acting as an antioxidant or a pro-oxidant agent, depending on the investigation context, such as the biological model and type of experiment carried out [36,67].

G18.EE and its fractions revealed a pro-oxidant activity in *BRAF*-mutated melanoma cells. Usually, ROS accumulation is linked to a pro-tumoral activity [68]. However, an overproduction of ROS can also be associated with antitumor activity, prompting ROS-mediated apoptosis, a type of regulated cell death [54]. Previous research has shown that ROS and p53 have a direct correlation [69]. The tumor suppressor protein p53 is activated by high levels of ROS, which subsequently activates the anti-apoptotic protein Bax and inhibits Bcl-2, a pro-apoptotic protein. During apoptosis, caspase-9 expression is also elevated. This caspase activates the effector caspase-3, causing the cleavage of cellular proteins and cell demise by apoptosis [69]. We looked at some specific apoptotic markers to see if there was a link between higher levels of ROS and activation of ROS-mediated apoptosis in melanoma cells. Caspase-9 (apoptosis-related protein) and Bcl-XL (anti-apoptotic protein) were significantly up and downregulated, respectively, in melanoma-treated cells (Figure 4). These outcomes support previous findings, indicating that G18.EE and its fractions (*n*-BuOH and EtOAc) trigger apoptosis in melanoma cells.

## 4. Materials and Methods

### 4.1. Propolis Sample

Propolis was collected in 2018 in an apiary close to the Cávado River, more precisely, between the settlements of Paradela and Sirvozelo, in Montalegre, Gerês, Portugal (41,045′41.62″ N; 7058′03.34″ W). Raw propolis was identified as G18, containing the capital letter G (from its origin: Gerês) followed by the last two digits of the harvesting year.

### 4.2. Preparation of Propolis Extract

Ethanol extraction of G18 was performed according to Freitas et al. [36]. Briefly, 80 mL of absolute ethanol were added to 15 g of raw propolis, which was previously ground into small pieces, and the mixture was kept under orbital agitation at 100 rpm for 24 h at 25 °C. The mixture was then filtered under vacuum (Macherey-Nagel filters), and the filtrate was stored at 4 °C in the dark, whereas the solid remains were subjected to a second extraction using 50 mL of absolute ethanol. Filtrates were pooled and the solvent completely evaporated in a Büchi Rotavapor RE 121 at 40 rpm and 38–40 °C, generating the dried ethanol extract G18.EE, which was stored at 4 °C, in the dark, until further use (yield of extraction = 61.63%).

Propolis solutions used in subsequent assays were prepared by diluting the dried extract in absolute ethanol for fractionation and DPPH and ABTS methods or dimethyl sulfoxide (DMSO) for in vitro assays.

### 4.3. Fractionation of Gerês Propolis from 2018 (G18)

Fractionation of propolis from Gerês was performed as previously described by Ana Freitas [62]. Briefly, 4 g of G18.EE was dissolved in 20 mL of absolute ethanol and, after becoming a homogenous solution, 200 mL of purified water were added. The mixture was successively partitioned with *n*-hexane, ethyl acetate (EtOAc), and *n*-butanol (3 × 400 mL each). Resulting organic phases were collected and dried over sodium anhydrous sulfate. *n*-Hexane, EtOAc, and *n*-butanol fractions were evaporated in a Büchi Rotavapor RE 121 at 40 rpm and 38–40 °C, generating the G18.EE-*n*-hexane, G18.EE-EtOAc, and G18.EE-*n*-BuOH dried fractions, respectively. The solvent used in each fraction was completely evaporated. All G18.EE-fractions were stored at 4 °C in the dark until further use and dissolved in DMSO to prepare stock solutions for further experiments.

### 4.4. Analysis of Phenolic Compounds from the Propolis Extract and Its Fractions

Total phenolic compounds from the ethanolic extract were estimated through the Folin–Ciocalteu method, as previously described [20]. The UPLC-DAD-ESI/MS^n^ analysis was performed as reported by Freitas et al. [36], using an Ultimate 3000 (Dionex Co., Sunnyvale, CA, USA) separation module equipped with an ultimate 3000 Diode Array Detector (DAD) (Dionex Co., Sunnyvale, CA, USA), and coupled to a mass spectrometer LTQ XL Linear Ion Trap 2D. The chromatographic system comprised a quaternary pump, an autosampler, a photodiode array detector, and an automatic thermostatic column compartment. The analysis was conducted on a Hypersil Gold (Thermo Scientific, Waltham, MA, USA) C18 column (100 mm length; 2.1 mm id; 1.9 μm particle diameter, end-capped), maintained at 30 °C. The mobile phase was composed of two phases: (A) 0.1% of formic acid (*v*/*v*) and (B) acetonitrile. The solvent gradient started with 20% of solvent (B), increasing to 40% at 25 min, 60% at 35 min, and 90% at 50 min, followed by the return to the initial conditions. The flow rate was 0.1 mL/min and UV-Vis spectral data for all peaks were acquired in the range of 200–500 nm, while the chromatographic profiles were recorded at 280 nm. The mass spectrometer used was a Thermo LTQ XL (Thermo Scientific, Waltham, MA, USA) ion trap MS equipped with an ESI source. The instrument was operated in negative-ion mode, with ESI needle voltage set at 5.00 kV and an ESI capillary temperature of 275 °C. The phenolic compounds were identified considering the retention times of standards and by comparison of the ESI-MS/MS with the data from MS/MS published in the literature.

### 4.5. Cell Lines and Culture Conditions

The *BRAF*^V600E^ mutant cell lines A375 and WM9 were established from malignant melanoma. A375 cell line was obtained from the American Type Culture Collection (ATCC^®^ CRL-1619^TM^), and WM9 cell line was kindly provided by Dr. Josane F. Sousa (University of São Paulo, Ribeirão Preto, Brazil) [70]. Melanoma cells were cultured in Dulbecco’s Modified Eagle’s Medium (DMEM; PAN-BIOTEC^TM^, Aidenbach, Germany) supplemented with 10% of Fetal Bovine Serum (FBS; PAN-BIOTEC^TM^, Aidenbach, Germany) (complete medium) and incubated at 37 °C in a humidified environment containing 5% of CO_2_.

### 4.6. Cell Viability Assay

Sulforhodamine B assay (SRB, TOX-6, Sigma-Aldrich, St. Louis, MO, USA) was used to assess the cell sensitivity of melanoma cell lines to the propolis ethanol extract and its fractions. A375 and WM9 cells were plated into 96-well plates at a concentration of 25 × 10^4^ cells/mL and allowed to adhere overnight in a complete medium. On the following day, plates were subjected to serum starvation for 2 h. The effect of propolis extract and its fractions, *n*-butanol, *n*-hexane, and EtOAc, was determined on cell number (total biomass) at 72 h (5 to 60 μg/mL) using DMEM supplemented with 0.5% of FBS. DMSO (control) and treatments were carried out at a final concentration of 0.1% DMSO. Triplicate wells were plated for each individual dose. After reaching the specific time point, the medium was discarded, and cells were fixed using 100 μL of cold 10% trichloroacetic acid (TCA) for 1 h at 4 °C. The cells were washed four times with de-ionized water and dried at 37 °C for 1.5 h. Then, 50 μL of SRB solution (0.4% SRB in 0.1% acetic acid) were added and incubated at RT for 30 min. After staining, washing was accomplished using 1% acetic acid (to eliminate unbound dye) and dried for 30 min at 37 °C until no liquid was evident. The dye was solubilized by adding 100 μL of 10 mM Tris base to each well, and plates were incubated for 10 min at RT. Absorbance was measured at 490 nm (Varioskan Flash, Thermo Scientific, Waltham, MA, USA). IC_15_, IC_25_, and IC_50_ values were calculated using GraphPad Software Version 8.0 [71,72]. Three independent experiments were carried out, each one conducted in triplicate.

After determining the IC_15_ and IC_25_ values, another SRB test was conducted to evaluate whether the concentrations of G18.EE and its fractions (*n*-butanol and EtOAc) were cytotoxic for the A375 and WM9 cells after 24 h, 48 h, and 72 h of treatment (5 to 13 g/mL). The methodology adopted was the same as the one described above, except for the treatment time. As previously, DMSO was used as control. Three independent assays were carried out, each one performed in triplicate.

### 4.7. Protein Extraction

A375 and WM9 cell lines were plated in six-well plates at a density of 20 × 10^4^ cells/well and allowed to adhere overnight in a complete medium. After 2 h of serum-starvation, cells were treated with 100 μg/mL, IC_15_ and IC_25_ concentrations of propolis ethanol extract (G18.EE) (10 and 13 μg/mL, respectively), *n*-butanol (5 and 7 μg/mL), and EtOAc (8 and 10 μg/mL) fractions, and with DMSO (control), for further 2 h. DMSO (control) and treatments were carried out at a final concentration of 0.1% DMSO. Protein was extracted by scraping the cells after adding lysis buffer (50 mM Tris pH 7.6–8, 150 mM NaCl, 5 mM EDTA, 1 mM NaOVa, 10 mM NaF, 10 mM NaPyrophosphatase, 1% NP-40 and 1:7 of Protease cocktail inhibitors (Roche^®^, Basel, Switzerland). Lysed cells were collected, kept on ice for 30 min, and centrifuged at 13,000 rpm, 4 °C for 15 min. The supernatant was collected, and protein was quantified using the Bradford reagent (Sigma-Aldrich, St. Louis, MO, USA) method. For quantification, 2 μL of protein extracts were added to each 96-well plate well, followed by 98 µL of PBS 1× and 200 µL of Bradford’s reagent. After a 5 min incubation period, absorbance was measured at 590 nm.

### 4.8. Western Blot

Aliquots of 40 μg of protein from A375 and WM9 cells were loaded onto a 15% polyacrylamide gel and separated by SDS-PAGE at 100 V. Protein was transferred onto a nitrocellulose membrane (Amersham Biosciences^®^, Amersham, United Kingdom) in 25 mM Tris-base/glycine buffer, as reported by Miranda-Gonçalves et al. [73]. To prevent unspecific binding, membranes were blocked with TBS-0.1% Tween containing 5% of BSA. To analyze the effect of G18.EE and its fractions on protein expression, membranes were incubated overnight at 4 °C with the primary polyclonal antibodies for Bax (1:2000, Rabbit, #2772, Cell Signaling Technology, Danvers, MA, USA), Bcl-2 (1:1000, Mouse, sc-7382, Santa Cruz Biotechnology, Dallas, TX, USA), Bcl-xL (1:1000, Mouse, #2764, Cell Signaling Technology, Danvers, MA, USA), Caspase 3 (1:1000, Rabbit, #9662, Cell Signaling Technology, Danvers, MA, USA), Caspase 9 (1:1000, Mouse, #9508, Cell Signaling Technology, Danvers, MA, USA), p53 (1:1000, Rabbit, #9282, Cell Signaling Technology, Danvers, MA, USA), NF-kB (1:1000, Rabbit, #8242, Cell Signaling Technology, Danvers, MA, USA), phospho-P53 (1:1000, Rabbit, #2521, Cell Signaling Technology, Danvers, MA, USA), and phosphor-NF-kB (1:1000, Rabbit, #3036, Cell Signaling Technology, Danvers, MA, USA). After incubation, membranes were washed in TBS 0.1% tween 3 × 5 min and then incubated with a secondary antibody coupled to horseradish peroxidase (1:2500, sc-2357 (Anti-rabbit) and sc-516102 (Anti-mouse), Santa Cruz Biotechnology, Dallas, TX, USA). Membranes were washed 2 × 5 min and 1 × 15 min with TBS-0.1% tween, and the bound antibodies were detected by chemiluminescence (WesternBright ECL HRP substrate, Advansta, San Jose, CA, USA) using Sapphire Biomolecular Imager (Azure Biosystems, Dublin, CA, USA). Tubulin was used as a loading control (1:2000, Rabbit, sc-6046, Santa Cruz Biotechnology, Dallas, TX, USA).

### 4.9. Evaluation of Antioxidant Activity of Propolis Extract and Its Fractions

#### 4.9.1. DPPH Radical Scavenging Activity

The DPPH (2,2-diphenyl-2-picry-lhydrazyl) colorimetric method was used to evaluate propolis scavenging activity [36,74]. Propolis extract was diluted in 100% ethanol (stock solution of 150 μg/mL) to yield final concentrations ranging from 0.5 to 50 μg/mL. Then, 50 μL of propolis solution were added to 100 μL of 0.004% (*w*/*v*) DPPH• and incubated at room temperature (RT), in the dark, for 20 min. Control was prepared with DPPH• and ethanol. Absorbance was measured at 517 nm, with ethanol used as blank. A solution of gallic acid (GA) was used as a standard, with concentrations ranging from 0.2 to 1.5 μg/mL. The EC_50_, defined as the concentration of an extract needed to scavenge 50% of the initial DPPH•, was calculated and expressed as the average value of three independent experiments with three replicates.

#### 4.9.2. ABTS Radical Scavenging Activity

To assess propolis scavenging activity, the 2,2′-azino-bis (3-ethylbenzothiazoline-6-sulfonic acid) (ABTS) cation radical decolorization experiment was carried out [52,75]. Propolis extract was diluted in 100% ethanol (stock solution of 150 μg/mL) to yield final concentrations ranging from 0.5 to 25 μg/mL; 7 mM ABTS aqueous solution was combined with 140 mM potassium persulphate for 14 to 16 h in the dark at room temperature to produce ABTS•. After that, the ABTS• working reagent was diluted in 100% ethanol, yielding a 734 nm absorbance of 0.70. Then, 2.5 μL of propolis solution was added to 247.5 μL of ABTS• working reagent and incubated for 30 min in the dark. Absorbance was measured at 734 nm against a blank prepared with 247.5 μL of 100% ethanol. A solution of Trolox was used as a standard. The EC_50_ was calculated and expressed as the average value of three independent experiments with three replicates.

#### 4.9.3. ROS Production and Mitochondrial Membrane Potential

The influence of G18.EE and its two fractions, *n*-butanol and EtOAc, on reactive oxygen species (ROS) production and mitochondrial activity were evaluated as previously described [76]. A375 and WM9 cell lines were plated at a density of 80 × 10^4^ cells/well and allowed to adhere overnight in a complete medium. The medium was discarded, and DMEM supplemented with 0.5% FBS was added. For both assays, cells were treated with DMSO (control), 100 μg/mL and IC_15_ and IC_25_ concentrations of propolis extract and its fractions. DMSO (control) and treatments were carried out at a final concentration of 0.1% DMSO. After 24 h of treatment, adherent cells were incubated with molecular probes for a period of 4 h at 37 °C in the dark. Ten millimoles of dihydroethidium (DHE, Molecular Probes, Eugene, OR, USA) was used to assess ROS production. For mitochondrial polarization and mitochondrial biomass, 50 nM of Mitotracker Red and 30 nM of Mitotracker Green (Molecular Probes, Eugene, OR, USA) were employed, respectively. Cells and respective supernatants were then collected into cytometry tubes and centrifuged for 5 min at 900 rpm and 4 °C. Cells were washed with PBS 1x and centrifuged again under the same conditions. Lastly, PBS 1× was removed, and 300 μL of Fluorescence-Activated Cell Sorting (FACs) was added to each flow cytometer tube. Analysis of ROS production and mitochondrial activity was performed by Flow Cytometry (BD LSR II). Assays were carried out in duplicate in three independent experiments.

### 4.10. Statistical Analysis

Results were expressed as mean ± standard deviation (SD) and statistically analyzed using the GraphPad Prism 8 software. Comparisons between different conditions were performed using the Two-way ANOVA test (Cell Viability Assay) and One-way ANOVA test (DHE and ROS and Mitochondrial Membrane potential). Significance was considered as *p* < 0.05.

## 5. Conclusions

G18.EE and its fractions showed cytotoxicity on *BRAF*-mutated A375 and WM9 cell lines, with *n*-BuOH being the most active fraction for both cell lines. This propolis fraction revealed a high proportion of CAPE, a known anticancer compound, suggesting it may be involved in the cytotoxic activity of Gerês propolis against melanoma as well.

The DPPH radical scavenging activity assay demonstrated antioxidant capacity for G18.EE. This bioactivity, however, was not confirmed in the melanoma context. In fact, G18.EE and its selected fractions (*n*-BuOH and EtOAc) increased ROS accumulation, mitochondrial activity, and activated apoptotic mechanisms, indicating pro-oxidant activity instead. Based on these findings, the DPPH assay seems insufficient to claim propolis as an antioxidant agent. Moreover, propolis from Gerês appears to have opposing activities depending on the experimental context.

To summarize, for the first time, we provided evidence for the anticancer activity of Portuguese propolis in melanoma. We suggest that this effect is mediated by a pro-oxidant mechanism involving the accumulation of ROS and activation of apoptotic pathways. Overall, the present study enabled the selection of *n*-BuOH as the G18.EE fraction with the highest activity against the most aggressive melanoma type (*BRAF*-mutated melanoma). This selection was crucial given the lower complexity of this fraction, facilitating the identification and further isolation of bioactive compounds. Thus, we believe that this natural product should continue to be explored as an important source of bioactive compounds with anticancer potential.

## Figures and Tables

**Table 1:** DPPH• and ABTS• scavenging activities of G18.EE. Results are expressed in EC_50_ (µg/mL) as mean ± standard deviation (SD). Gallic acid and Trolox were used as standards for the DPPH and ABTS assays, respectively.

	DPPH•	ABTS (Absolute Ethanol)
	EC_50_ (µg/mL)	EC_50_ (µg/mL)
G18.EE	10.90 ± 0.34	9.83 ± 0.21
Gallic Acid	1.21 ± 0.08	- - - - -
Trolox	- - - - -	3.46 ± 0.22

**Table 2:** IC_50_, IC_25_, and IC_15_ values of the Portuguese propolis ethanol extract under study (G18.EE) and respective fractions (*n*-hexane, EtOAc, and *n*-BuOH) against melanoma cell lines. A375 and WM9 cells were treated for 72 h with 5 to 60 µg/mL of each fraction. Results are expressed as mean ± SD.

	IC_50_ (µg/mL)		IC_25_ (µg/mL)		IC_15_ (µg/mL)	
	A375	WM9	A375	WM9	A375	WM9
G18.EE	16.98 ± 0.93	25.03 ± 1.34	10.85 ± 0.12	15.32 ± 0.14	8.88 ± 0.12	12.05 ± 0.14
*n*-hexane	45.71 ± 1.69	39.54 ± 0.17	24.79 ± 0.09	20.49 ± 0.04	19.13 ± 0.11	15.48 ± 0.04
EtOAc	17.12 ± 0.72	16.39 ± 0.46	12.8 ± 0.03	8.56 ± 0.11	10.9 ± 0.09	6.69 ± 0.15
*n*-BuOH	8.14 ± 0.03	11.22 ± 1.66	6.16 ± 0.10	8.08 ± 0.22	4.57 ± 0.09	6.01 ± 0.11

**Table 3:** IC_15_ and IC_25_ intermediate values selected for G18.EE and *n*-BuOH and EtOAc fractions against melanoma cells.

	IC_15_ (µg/mL)	IC_25_ (µg/mL)
G18.EE	10	13
EtOAc	8	10
*n*-BuOH	5	7

## Data Availability

Data is contained within the article.

## Supplementary Materials

The following supporting information can be downloaded at: https://www.mdpi.com/article/10.3390/molecules27113533/s1, Figure S1: Chromatographic profiles at 280 nm of G18.EE and its fractions *n*-hexane, EtOAc, and *n*-BuOH, obtained by LC-MS; Table S1: Chemical composition of G18.EE and its fractions *n*-hexane, EtOAc, and *n*-BuOH according to LC-MS analysis; Table S2: Analysis of the effect of the selected propolis fractions’ treatments on cell biomass through time; Table S3: Analysis of the effect of the selected propolis fractions’ treatments on melanoma cells ROS levels; Table S4: Analysis of the effect of the selected propolis fractions’ treatments on mitochondrial activity; Table S5: Analysis of the effect of the selected propolis fractions’ treatments on mitochondrial biomass; Table S6: Analysis of the effect of selected propolis fractions’ treatments on mitochondrial polarization; Table S7: Analysis of the effect of propolis fractions’ treatments on the levels of apoptotic markers.

## Sample Availability

Samples of the compounds are not available from the authors.
