# Supplementary material for: Portuguese Propolis Antitumoral Activity in Melanoma Involves ROS Production and Induction of Apoptosis
Source: Molecules. 2022 May 31;27(11):3533. doi: 10.3390/molecules27113533 (PMC9182411; doi:10.3390/molecules27113533)
Supplement: Supplementary file 1 [file molecules-27-03533-s001.zip › molecules-1660302-supplementary.pdf]

Supplementary Material

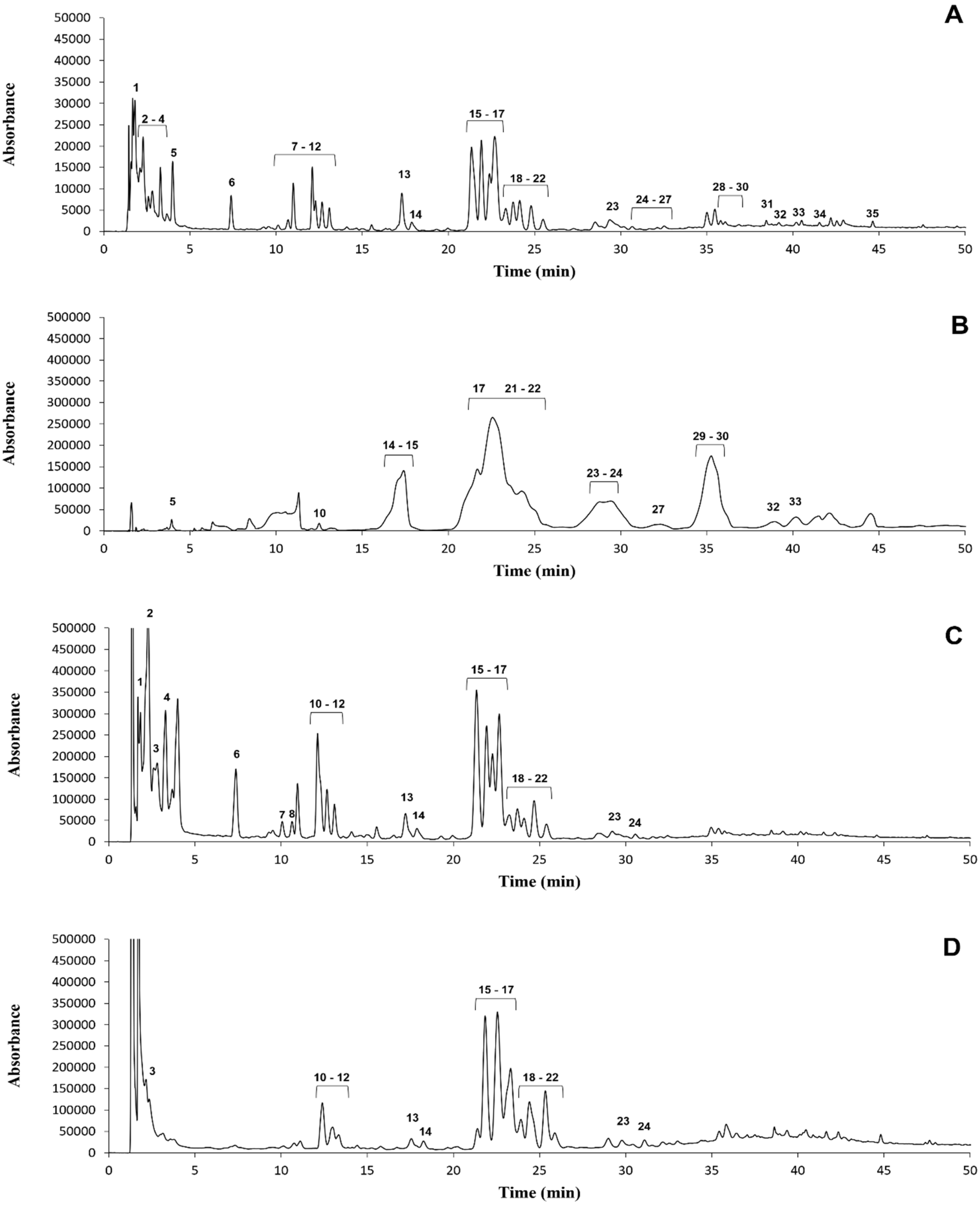

Figure S1 - Chromatographic profiles of G18.EE (A) and its fractions η-Hexane (B), EtOAc (C), and η-BuOH (D) obtained by LC-MS.

Table S1 - Chemical composition of G18.EE and its fractions η-Hexane, EtOAc, and η-BuOH according to LC-MS analysis. (“-“corresponds to a compound not present or not detected). Bold values represent the major phenolic compounds (Composition (%) > 5%).

Peaks	Time range (min)	λmax (nm)	$[M - H]^-$ m/z	Main fragments	Compound	Composition (%)			
						G18.EE	η-Hexane	EtOAc	η-BuOH
**1**	1.6 – 2.2	270	169	125	Gallic acid	3.39	-	1.65	-
**2**	2.2 – 2.5	292sh, 322	179	135, 163	Caffeic acid	**7.08**	-	**14.97**	-
**3**	2.3 – 3.6	253, 368	301	257, 229	Ellagic acid	2.15	0.05	0.74	2.64
**4**	3.1 – 3.5	309	163	119, 145, 108	*p*-Coumaric acid	3.33	-	**7.83**	-
**5**	3.8 – 4.2	295sh, 323	193	177, 149, 133	Ferulic acid	3.97	0.45	-	-
**6**	7.3 – 7.6	295sh, 322	207	163, 148, 133	3,4-Dimethyl-caffeic acid (DMCA)	2.15	-	6.31	-
**7**	9.9 – 10.3	288	285	267, 239, 252	Pinobanksin-5-methyl-ether	0.49	-	1.30	-
**8**	10.5 – 10.8	273, 353	315	300	Quercetin-3-methyl ether	0.84	-	1.16	-
**9**	10.8 – 11.2	308	177	-	*p*-coumaric acid methyl ester	2.72	-	-	-
**10**	11.9 – 12.7	267, 336<br>291	269<br>271	225, 151<br>253, 225	Apigenin<br>Pinobanksin	**5.03**	0.64	**10.14**	**7.39**
**11**	12.5 – 13.2	265, 363	285	285, 257, 151	Kaempferol	2.01	-	2.87	3.04
**12**	13.0 – 13.6	255, 368	315	300	Isorhamnetin	1.32	-	2.35	1.18
**13**	16.7 – 17.9	311	313	298, 269, 257, 241, 179, 153	unknown	3.30	4.16	2.47	2.66
**14**	17.3 – 18.5	254, 368	329	-	Quercetin-dimethyl-ether	1.01	1.11	1.12	1.52
**15**	20.5 – 22.4	268, 315<br>298, 325	253<br>247	209, 181, 225, 151<br>179, 135	Chrysin<br>Caffeic acid isoprenyl ester	**10.35**	**28.99**	**15.93**	**20.94**
**16**	21.7 – 23.1	268, 332<br>299, 325	283<br>247	268<br>179, 135	Acacetin<br>Caffeic acid isoprenyl ester	**12.73**	-	**11.48**	**23.43**
**17**	22.5 – 23.6	289	255	213, 211, 151	Pinocembrin	**13.01**	**20.79**	**8.24**	**5.52**

Table S1 (continued) - Chemical composition of G18.EE and its fractions η-Hexane, EtOAc, and η-BuOH according to LC-MS analysis. ("-" corresponds to a compound not present or not detected). Bold values represent the major phenolic compounds. (Composition (%) > 5%).

Peaks	Time range (min)	λmax (nm)	[M - H]- m/z	Main fragments	Compound	Composition (%)			
						G18.EE	η-Hexane	EtOAc	η-BuOH
**18**	23.0 – 24.2	266, 290sh, 356	269	223, 169, 249	Galangin	2.69	-	1.35	2.95
**19**	23.6 – 24.5	267, 362	299	284, 165	Kaempferide	2.72	-	1.93	4.38
**20**	24.0 – 25.1	269, 360	329	314, 297, 287, 269, 257	Kaempferol –methoxy-methyl ether	3.13	-	1.02	2.29
**21**	23.9 – 25.8	293	313	253, 271	Pinobanksin-3-*O*-acetate	2.56	4.38	3.82	9.75
**22**	25.2 – 29.7	298, 325	283	179, 135	Caffeic acid phenylethyl ester	1.39	0.41	1.55	2.24
**23**	27.6 – 30.9	311	231	163, 119	*p*-Coumaric acid isoprenyl ester (isomer)	2.00	3.49	0.50	1.64
**24**	30.3 – 31.4	295, 325	295	178, 134, 251, 211	Caffeic acid cinnamyl ester	0.58	0.53	0.39	1.34
**25**	31.5 – 31.8	268, 305b	297<br>403	179, 161, 151, 135<br>-	Caffeic acid derivative<br>unknown	0.29	-	-	-
**26**	32.3 – 32.8	293	327	253, 271	Pinobanksin-3-O-propionate	0.32	-	-	-
**27**	31.6 – 34.4	340	269	254, 251, 236, 165	3-Hydroxy-5-methoxy flavanone	0.65	1.78	0.25	1.04
**28**	35.7 – 35.9	268, 309b	387<br>501	281, 267, 255, 293<br>457, 439, 247	unknown<br>unknown	2.09	-	-	-
**29**	34.3 – 36.5	290	417	-	Methylated Pinobanksin-3-*O*-phenylpropionate	0.93	**19.03**	0.16	2.52
**30**	35.5 – 39.4	293	341	253	Pinobanksin-3-*O*-butyrate or isobutyrate	0.48	**8.11**	0.04	0.95
**31**	38.5 – 40.3	269, 307, 343	565	283, 269, 281, 417, 455	*p*-Coumaric acid-4-hydroxyphenylethyl ester dimer	0.87	-	-	1.09
**32**	38.1 – 41.8	292	355	253, 271	Pinobanksin-3-*O*-pentenoate or 2-methylbutyrate	0.89	3.24	0.21	0.97
**33**	39.6 – 44.8	297, 320b	315	179, 135	Caffeic acid derivative	0.98	2.82	0.24	0.53

Table S1 (conclusion) - Chemical composition of G18.EE and its fractions η-Hexane, EtOAc, and η-BuOH according to LC-MS analysis. ("-" corresponds to a compound not present or not detected). Bold values represent the major phenolic compounds. (Composition (%) > 5%).

Peaks	Time range (min)	λmax (nm)	[M - H]⁻ m/z	Main fragments	Compound	Composition (%)			
						G18.EE	η-Hexane	EtOAc	η-BuOH
**34**	41.4 – 41.7	280	293	185, 197, 275, 249	*p*-Methoxy-cinnamic acid cinnamyl ester	0.85	-	-	-
**35**	44.5 – 44.8	310	473	-	*p*-Coumaric acid derivative	0.85	-	-	-

Table S2 - Analysis of the effect of the selected propolis fractions' treatments on cell biomass along the time. Statistical analyses were performed with Two-way ANOVA for both cell lines (A375 and WM9). Bold p-values show statistically significant effects.

**A375 Cell Line**						
**Source of Variation**	**Sum of Squares**	**Degrees of Freedom**	**Mean Squares**	**F ratio**	***p*-value**	**Partial Eta Squared**
**Time**	31319	3	10440	F(2.096,20.96) = 72.40	**<0.0001**	0.879
**Treatment**	3404	4	850.9	F(4,10) = 4.774	**0.0205**	0.440
**Time vs Treatment**	1494	12	124.5	F(12,30) = 0.8633	0.5901	0.257
**Residual**	4326	30	144.2	- - - - -	- - - - -	- - - - -

**WM9 Cell Line**						
**Source of Variation**	**Sum of Squares**	**Degrees of Freedom**	**Mean Squares**	**F ratio**	***p*-value**	**Partial Eta Squared**
**Time**	4413	3	1471	F(1.192,11.92) = 15.83	**0.0013**	0.613
**Treatment**	7092	4	1773	F(4,10) = 2.344	0.1252	0.718
**Time vs Treatment**	3330	12	277.5	F(12,30) = 2.986	**0.0074**	0.544
**Residual**	2788	30	92.94	- - - - -	- - - - -	- - - - -

vs = versus

Table S3 - Analysis of the effect of the selected propolis fractions' treatments on melanoma cells' ROS levels. Statistical analyses were performed with One-way ANOVA for both cell lines (A375 and WM9). Bold p-values show statistically significant effects.

			A375 Cell Line			
**Source of Variation**	**Sum of Squares**	**Degrees of Freedom**	**Mean Squares**	**F ratio**	***p*-value**	**Partial Eta Squared**
**Treatment**	52679542	6	8779924	F(6,14) = 59.85	**<0.0001**	0.9625
**Residual**	2053646	14	146689	- - - - -	- - - - -	- - - - -
			**WM9 Cell Line**			
**Source of Variation**	**Sum of Squares**	**Degrees of Freedom**	**Mean Squares**	**F ratio**	***p*-value**	**Partial Eta Squared**
**Treatment**	41822810	6	6970468	F(6,14) = 3.972	**0.0157**	0.6299
**Residual**	24570069	14	1755005	- - - - -	- - - - -	- - - - -

Table S4 - Analysis of the effect of the selected propolis fractions' treatments on mitochondrial activity. Statistical analyses were performed with One-way ANOVA for both cell lines (A375 and WM9).

			A375 Cell Line			
**Source of Variation**	**Sum of Squares**	**Degrees of Freedom**	**Mean Squares**	**F ratio**	***p*-value**	**Partial Eta Squared**
**Treatment**	51.67	6	8.612	F(6,14) = 2.467	0.0769	0.5139
**Residual**	48.88	14	3.491	- - - - -	- - - - -	- - - - -
			**WM9 Cell Line**			
**Source of Variation**	**Sum of Squares**	**Degrees of Freedom**	**Mean Squares**	**F ratio**	***p*-value**	**Partial Eta Squared**
**Treatment**	8.320	6	1.387	F (6, 14) = 2.176	0.1082	0.4826
**Residual**	8.921	14	0.6372	- - - - -	- - - - -	- - - - -

Table S5 - Analysis of the effect of the selected propolis fractions' treatments on mitochondrial biomass. Statistical analyses were performed with One-way ANOVA for both cell lines (A375 and WM9). Bold p-values show statistically significant effects.

**A375 Cell Line**						
**Source of Variation**	**Sum of Squares**	**Degrees of Freedom**	**Mean Squares**	**F ratio**	***p*-value**	**Partial Eta Squared**
**Treatment**	10580118	6	1763353	F(6,14) = 5.993	**0.0028**	0.7198
**Residual**	4119184	14	294227	- - - - -	- - - - -	- - - - -
**WM9 Cell Line**						
**Source of Variation**	**Sum of Squares**	**Degrees of Freedom**	**Mean Squares**	**F ratio**	***p*-value**	**Partial Eta Squared**
**Treatment**	22875260	6	3812543	F (6, 14) = 10.33	**0.0002**	0.8158
**Residual**	5165729	14	368981	- - - - -	- - - - -	- - - - -

Table S6 - Analysis of the effect of selected propolis fractions' treatments on mitochondrial polarization. Statistical analyses were performed with One-way ANOVA for both cell lines (A375 and WM9).

**A375 Cell Line**						
**Source of Variation**	**Sum of Squares**	**Degrees of Freedom**	**Mean Squares**	**F ratio**	***p*-value**	**Partial Eta Squared**
**Treatment**	9159783	6	1526631	F(6,14) = 2.662	0.0616	0.5329
**Residual**	8030032	14	573574	- - - - -	- - - - -	- - - - -
**WM9 Cell Line**						
**Source of Variation**	**Sum of Squares**	**Degrees of Freedom**	**Mean Squares**	**F ratio**	***p*-value**	**Partial Eta Squared**
**Treatment**	3083553	6	513926	F (6, 14) = 1.248	0.3407	0.3485
**Residual**	5763906	14	411708	- - - - -	- - - - -	- - - - -

Table S7 - Analysis of the effect of propolis fractions' treatments on the levels of apoptotic markers. Statistical analyses were performed with One-way ANOVA for both cell lines (A375 and WM9). Bold p-values show statistically significant effects.

A375 Cell Line – Bax protein						
**Source of Variation**	**Sum of Squares**	**Degrees of Freedom**	**Mean Squares**	**F ratio**	***p*-value**	**Partial Eta Squared**
**Treatment**	0.4133	4	0.1033	F(4,10) = 2.431	0.1161	0.4930
**Residual**	0.4250	10	0.04250	- - - - -	- - - - -	- - - - -
**WM9 Cell Line – Bax protein**						
**Source of Variation**	**Sum of Squares**	**Degrees of Freedom**	**Mean Squares**	**F ratio**	***p*-value**	**Partial Eta Squared**
**Treatment**	0.4526	4	0.1131	F(4,10) = 0.6521	0.6385	0.2069
**Residual**	1.735	10	0.1735	- - - - -	- - - - -	- - - - -
**A375 Cell Line – Bcl-2 protein**						
**Source of Variation**	**Sum of Squares**	**Degrees of Freedom**	**Mean Squares**	**F ratio**	***p*-value**	**Partial Eta Squared**
**Treatment**	0.3866	4	0.09666	F(4,10) = 0.6709	0.6269	0.2115
**Residual**	1.441	10	0.1441	- - - - -	- - - - -	- - - - -
**WM9 Cell Line – Bcl-2 protein**						
**Source of Variation**	**Sum of Squares**	**Degrees of Freedom**	**Mean Squares**	**F ratio**	***p*-value**	**Partial Eta Squared**
**Treatment**	0.1520	4	0.03800	F(4,10) = 0.4382	0.7785	0.1491
**Residual**	0.8673	10	0.08673	- - - - -	- - - - -	- - - - -
**A375 Cell Line – Caspase 3 protein**						
**Source of Variation**	**Sum of Squares**	**Degrees of Freedom**	**Mean Squares**	**F ratio**	***p*-value**	**Partial Eta Squared**
**Treatment**	0.3043	4	0.07606	F(4,10) = 2.131	0.1514	0.4644
**Residual**	0.3510	10	0.03570	- - - - -	- - - - -	- - - - -
**WM9 Cell Line – Caspase 3 protein**						
**Source of Variation**	**Sum of Squares**	**Degrees of Freedom**	**Mean Squares**	**F ratio**	***p*-value**	**Partial Eta Squared**
**Treatment**	0.3399	4	0.08498	F(4,10) = 0.9050	0.4969	0.2658
**Residual**	0.9390	10	0.09390	- - - - -	- - - - -	- - - - -
**A375 Cell Line – Caspase 9 protein**						
**Source of Variation**	**Sum of Squares**	**Degrees of Freedom**	**Mean Squares**	**F ratio**	***p*-value**	**Partial Eta Squared**
**Treatment**	0.5295	4	0.1324	F(4,10) = 4.811	**0.0201**	0.6581
**Residual**	0.2751	10	0.02751	- - - - -	- - - - -	- - - - -
**WM9 Cell Line – Caspase 9 protein**						
**Source of Variation**	**Sum of Squares**	**Degrees of Freedom**	**Mean Squares**	**F ratio**	***p*-value**	**Partial Eta Squared**
**Treatment**	2.920	4	0.7299	F(4,10) = 12.98	**0.0006**	0.8385
**Residual**	0.5623	10	0.05623	- - - - -	- - - - -	- - - - -

Table S7 (continued) - Analysis of the effect of propolis fractions' treatments on the levels of apoptotic markers. Statistical analyses were performed with One-way ANOVA for both cell lines (A375 and WM9). Bold p-values show statistically significant effects.

**A375 Cell Line – Bcl-XL protein**						
**Source of Variation**	**Sum of Squares**	**Degrees of Freedom**	**Mean Squares**	**F ratio**	***p*-value**	**Partial Eta Squared**
**Treatment**	1.937	4	0.4843	F(4,10) = 10.31	**0.0014**	0.8048
**Residual**	0.4697	10	0.04697	- - - - -	- - - - -	- - - - -
**WM9 Cell Line – Bcl-XL protein**						
**Source of Variation**	**Sum of Squares**	**Degrees of Freedom**	**Mean Squares**	**F ratio**	***p*-value**	**Partial Eta Squared**
**Treatment**	0.02661	4	0.006653	F(4,10) = 0.3726	0.8231	0.1298
**Residual**	0.1786	10	0.01786	- - - - -	- - - - -	- - - - -
**A375 Cell Line – p53 protein**						
**Source of Variation**	**Sum of Squares**	**Degrees of Freedom**	**Mean Squares**	**F ratio**	***p*-value**	**Partial Eta Squared**
**Treatment**	0.5521	4	0.1380	F(4,10) = 1.667	0.2331	0.4000
**Residual**	0.8281	10	0.08281	- - - - -	- - - - -	- - - - -
**WM9 Cell Line – p53 protein**						
**Source of Variation**	**Sum of Squares**	**Degrees of Freedom**	**Mean Squares**	**F ratio**	***p*-value**	**Partial Eta Squared**
**Treatment**	0.8925	4	0.2231	F(4,10) = 1.426	0.2949	0.3632
**Residual**	1.565	10	0.1565	- - - - -	- - - - -	- - - - -
